# Anemoside B4 ameliorates TNBS-induced colitis through S100A9/MAPK/NF-κB signaling pathway

**DOI:** 10.1186/s13020-020-00410-1

**Published:** 2021-01-18

**Authors:** Yong Zhang, Zhengxia Zha, Wenhua Shen, Dan Li, Naixin Kang, Zhong Chen, Yanli Liu, Guoqiang Xu, Qiongming Xu

**Affiliations:** 1 College of Pharmaceutical Sciences, Soochow University, Suzhou, 215123 Jiangsu China; 2 Jiangsu Key Laboratory of Neuropsychiatric Diseases and Jiangsu Key Laboratory of Preventive and Translational Medicine for Geriatric Diseases, Soochow University, Suzhou, 215123 Jiangsu China

**Keywords:** Anemoside B4, Colitis, Quantitative proteomics, S100A9, NF-κB

## Abstract

**Background:**

Despite the increased morbidity of ulcerative colitis (UC) in the developing countries, available treatments remain unsatisfactory. Therefore, it is urgent to discover more effective therapeutic strategies. *Pulsatilla chinensis* was widely used for the treatment of inflamed intestinal diseases including UC for thousands of years in China. Anemoside B4, the most abundant triterpenoid saponin isolated from *P. chinensis*, exerts anti-inflammatory and antioxidant effects and may be the most active compounds, which is responsible for the therapeutic effects. However, the mechanism how anemoside B4 executes its biological functions is still elusive.

**Methods:**

Here, we used the 2, 4, 6-trinitrobenzene sulfonic acid (TNBS)-induced colitis rat model to evaluate the therapeutic effect of anemoside B4. Blood samples of colitis rats were collected for hematology analysis. The inflammation-associated factors were investigated by enzyme-linked immunosorbent assay (ELISA). Cell proliferation and apoptosis was determined with EdU cell proliferation assay and TUNEL assay. The proteins regulated by anemoside B4 were identified by label-free quantitative proteomics. The significantly down-regulated proteins were verified by Western blotting analysis. mRNA expression was analyzed by quantitative real-time RT-PCR.

**Results:**

The results showed that anemoside B4 ameliorated TNBS-induced colitis symptoms, including tissue damage, inflammatory cell infiltration, and pro-inflammatory cytokine production, apoptosis and slowed proliferation in colon. Quantitative proteomic analyses discovered that 56 proteins were significantly altered by anemoside B4 in the TNBS-induced rats. These proteins mainly clustered in tricarboxylic acid (TCA) cycle and respiratory electron transport chain. Among the altered proteins, S100A9 is one of the most significantly down-regulated proteins and associated with NF-κB and MAPK signaling pathways in the pathogenesis of UC. Further experiments revealed that anemoside B4 suppressed the expression of S100A9 and its downstream genes including TLR4 and NF-κB in colon. In vitro, anemoside B4 could inhibit the NF-κB signaling pathway induced by recombinant S100A9 protein in human intestinal epithelial Caco-2 cells. Moreover, anemoside B4 inhibits neutrophils recruitment and activation in colon induced by TNBS.

**Conclusions:**

Our results demonstrate that anemoside B4 prevents TNBS-induced colitis by inhibiting the NF-κB signaling pathway through deactivating S100A9, suggesting that anemoside B4 is a promising therapeutic candidate for colitis.

## Introduction

Inflammatory bowel disease (IBD) is a prevailing disease worldwide, especially in developing countries, and its incidence has increased significantly in the past two decades [1]. The two main forms of IBD are ulcerative colitis (UC) and Crohn’s disease (CD), which are characterized by abdominal pain, diarrhea, bowel obstruction, weight loss, and associated immune disorders. Both of them are incurable and usually diagnosed at a young age with a significant morbidity [2].

It is currently accepted that multiple factors, such as microbial flora, dysregulation of the immune response, genetic susceptibility, and environmental factors, are involved in IBD, although its precise etiology remains elusive. In particular, a pronounced imbalance in the activation of pro-inflammatory and anti-inflammatory signaling pathways in gut is thought to be an important contributor to the development and progression of IBD.

Current clinical treatments for UC include several anti-inflammatory drugs, such as sulfasalazine, glucocorticoids, nonsteroid anti-inflammatory agents, inhibitors of pro-inflammatory pathways, tumor necrosis factor (TNF)-α, gut-homing α4β7 integrin, interleukin (IL)-12/IL-23, and Janus kinases [3]. However, these drugs are less effective for some patients and frequently cause severe side effects, including opportunistic infections and malignancies [4]. Therefore, no effective and safe clinical treatment has been discovered for IBD patients.

At present, the use of complementary and alternative medicine, including acupuncture, homeopathy and herbal medicines, for the treatment of patients with IBD is increasing. It is estimated that up to 70% of patients with IBD in North America and Europe were treated with complementary and alternative medicine [5, 6]. In China, several Chinese herbs are frequently used in the treatment of IBD, such as *Pulsatilla chinensis* (Bunge) Regel and Pulsatilla decoction, which were mainly used to treat UC in the traditional Chinese medicine for thousands of years [7]. Pulsatilla decoction possesses a variety of pharmacological effects and the active ingredients from these herbal plants have exhibited hepatic protective, antiinflammatory, antibacterial, anti-tumor and antioxidant effects [8]. Several studies have reported that Pulsatile decoction can alleviate the release of inflammatory factors, such as IL-17, IL-1β and TNF-α in the serum of IBD patients, reduce syndromes and symptom scores, and suppress the activation of the NF-κB signaling pathway [9].

As the most important herb in factors decoction, *P. chinensis* exhibits “blood-cooling” and detoxification activities and has been widely used for the treatment of amoebic dysentery and malignant dysentery in China for two thousand years [10]. Accumulating evidence has demonstrated that *P. chinensis* and its ingredients have antioxidant, anti-inflammatory and anti-tumor effects. Our previous study reported that anemoside B4, the most abundant triterpenoid saponin isolated from *P. chinensis*, reduces inflammation and oxidant injury induced by cisplatin. It ameliorates LPS-induced kidney and lung damage, inhibits LPS-induced NF-κB activation in vivo [11]. Recently, many studies have been conducted to investigate the anti-inflammatory, immunoregulatory and anti-tumor activities of anemoside B4 with acceptable safety profile after acute i.v. or subacute i.p. injection [12–16]. Considering the major application of Bai-Tou-Weng-Tang for the treatment of UC and the high content of anemoside B4 in Bai-Tou-Weng-Tang together with its better distribution and ease of study in the intestine of colitis animals [17], it may be a more potent anti-UC agent than berberine as discovered in previous studies [14]. However, the molecular mechanism of action of anemoside B4 is unclear.

In this study, we investigated the therapeutic effect of anemoside B4 on 2, 4, 6-trinitrobenzene sulfonic acid (TNBS)-induced colitis in rats. We found that anemoside B4 exerted anti-inflammatory and anti-apoptotic properties through down-regulating colonic inflammatory cytokine levels and inhibiting colonic epithelium apoptosis. Quantitative proteomic analyses found that 56 proteins were significantly altered by anemoside B4 in the colon of colitis rats. Among the altered proteins, S100A9 is the most significantly down-regulated protein which is associated with NF-κB and MAPK signaling pathways in the pathogenesis of UC. Furthermore, we revealed that anemoside B4 prevented colitis in rats by inhibiting the NF-κB signaling pathway through inactivating S100A9.

## Methods

### Chemicals and reagents

TNBS (Cat #: P2297) and lipopolysaccharide (LPS) were supplied by Sigma-Aldrich, Germany (L6529-1MG). Mesalazine (89-57-6) was purchased from Ethypharm Pharmaceutical Co Ltd (Shanghai, China). Human recombinant S100A9 protein was purchased from Abcam (AB95909). Rat IL-1β, IL-6, and TNF-α ELISA kits were supplied by MultiSciences (Hangzhou, China). PCR primers for quantitative real-time PCR analysis were synthesized by GeneWiz (Suzhou, China). The primary antibodies for p38 (8960, 1:2000 for Western blotting), p-p38 (4511, 1:1000), JNK (9252, 1:1000), p-JNK (9255, 1:2000), ERK1/2 (4695, 1:2000), p-ERK1/2 (4370, 1:1000), NF-κB/p65 (8242, 1:2000), p-NF-κB/p65 (3033, 1:1000), Bcl-2 (3498, 1:1000), cleaved-caspase 3 (9664, 1:1000), and S100A9 (73,425, 1:1000) were purchased from Cell Signaling Technology (Danvers, USA), and Bax (sc-20,067, 1:2000), p53 (sc-126, 1:1000), IL-6 (sc-57,315, 1:1000), TLR4 (sc-293,072, 1:1000), NOD2 (sc-56,168, 1:1000), and GAPDH (sc-365,062, 1:2000) were purchased from Santa Cruz Biotechnology Co. Ltd (Santa Cruz, USA). Anemoside B4 (20 g, purity more than 99%) was isolated from the roots of *P. chinensis* and its structure was determined as previous described [11].

### Experimental animals

Sprague-Dawley (SD) rats (male, 220–250 g) were obtained from the Experimental Animal Center of Soochow University. All animals were given free access to water and food with air filtration (22 ± 2 °C, 12-h light/12-h dark). All animal experiments were conducted in accordance with the procedure approved by the Ethical Review Committee for Laboratory Animal Welfare of the Soochow University.

### Induction of colitis and experimental design

All animals were divided into five groups (n = 7 per group): vehicle group, TNBS + saline group, TNBS + anemoside B4 group (5 mg/kg), TNBS + anemoside B4 group (10 mg/kg), and TNBS + mesalazine group (200 mg/kg). All rats were fasted for 24 h and weighed before injection, and then were anesthetized by chloral hydrate intraperitoneally (300 mg/kg, i.p.). In the vehicle group, 40% ethanol was slowly instilled into the colon of anesthetized rats for 1 min, while for the other four groups, TNBS solution (80 mg/kg in 40% ethanol v/v) was instilled for 1 min. Rats in the vehicle group and TNBS + saline group were intraperitoneally injected with saline, and those in the TNBS + anemoside B4 groups were intraperitoneally injected with anemoside B4, while those in the TNBS + mesalazine group were treated with mesalazine by gavage once per day for 7 days. Anemoside B4 (5 mg/kg, 10 mg/kg) or saline was injected twice per day for 7 days after treatment with TNBS for 24 h.

### Assessment of colonic damage

During the experiment, rats were weighed every day and disease activity index (DAI) was recorded for seven days after the induction of colitis according to previous reference [18]. The DAI is the combined score of weight loss compared to initial weight, stool consistency, and bleeding. In brief, no weight loss was registered as 0, weight loss of 1% to 5% from baseline was assigned 1 point, weight loss of 6% to 10% from baseline was assigned 2 points, weight loss of 11% to 20% from baseline was assigned 3 points and weight loss of more than 20% from baseline was assigned 4 points. Stool consistency scores were 0 (normal, well-formed pellets), 2 (loose stool, pasty and semi-formed stools that did not adhere to the anus), and 4 (diarrhea, liquid stools that adhered to the anus). Bleeding scores were 0 (no blood), 2 (Hemoccult positive and visual pellet bleeding), and 4 (gross bleeding, blood around anus).On the 8th day, rats were sacrificed by anesthesia. The colons were harvested and rinsed with saline. Colon length was recorded as an indirect marker of inflammation. Myeloperoxidase (MPO) activity was measured to assess inflammation. Colon samples were fixed in 4% paraformaldehyde for histological examination and immunofluorescence staining. The remaining colon samples were kept at -80 °C for further assessment by quantitative proteomics, enzyme-linked immunosorbent assay (ELISA), qPCR, and Western blotting.

### Histopathological analysis of rectums

For histological assessments, sections of rectums with a thickness of 5 µm were stained with hematoxylin and eosin (H&E) to visualize cell morphology using an optical microscope. Light microscopy (CX31 research microscope, Olympus Optical Co. Ltd., Tokyo, Japan) and panoramic viewer camera system were used to examine, scan, and analyze the sections. Histological score was measured by inflammation severity: 0 (normal colonic mucosal), 1 (crypt damage less than 1/3), 2 (crypt damage less than 1/3−2/3), 3 (mucosal erosion), 4 (muscosal erosion or ulcer with significant infiltration of inflammatory cells) according to a previous reference [19].

### Label-free quantitative proteomics

On the 8th day after rats were treatment with TNBS (80 mg/kg) and/or anemoside B4, colon tissues from the rats in TNBS + saline group (n = 3) and TNBS + anemoside B4 group (10 mg/kg) (n = 3) were extracted, lysed in a HEPES buffer (pH 8.0) containing 8 M urea to obtain total proteins. Disulfide bonds in these proteins were reduced with 2 mM dithiothreitol at 37 °C for 45 min. The free thiols were further alkylated with 8 mM chloroacetamide for 1 h at room temperature. The excess chloroacetamide was then quenched by adding 2 mM dithiothreitol for 45 min. After acetone precipitation, proteins were resuspended in a HEPES buffer containing 8 M urea and incubated with the MS sequencing grade Lys-C (TaKaRa Bio, Japan) for 4 h at 37 °C. Then the samples were diluted with 10 mM HEPES (pH 8.0) four times and continued for digestion with MS sequencing grade trypsin (TaKaRa Bio, Japan) at 37 °C for 20 h. Next, the peptides were desalted with C_18_ Zip-tips (Merck Millipore, MA, USA), concentrated, separated by capillary high performance liquid chromatography and analyzed in an Orbitrap Fusion Lumos mass spectrometer (Thermo Fisher Scientific, MA, USA). Data were analyzed using MaxQuant according to a previous procedure [20]. Proteins with FDR ≤ 0.01 were considered as positive identification. The independent sample *t*-test in IBM SPSS software (Ver 19) was used to calculate the *P*-values for the identified proteins. The proteins with fold change > 1.50 or < 0.67 and *P* < 0.05 were considered as significantly differentially expressed proteins.

### Western blotting analysis

Western blotting analysis was performed as previously described [11]. In brief, proteins from tissues or cells were extracted in ice-cold RIPA buffer supplemented with protease inhibitor mixture (Thermo Fisher Scientific, Germany), separated by SDS-PAGE, and transferred into PVDF membranes. After blocking with 5% bovine serum albumin for 1 h at room temperature, membranes were incubated overnight at 4 °C with the following primary antibodies: p38, p-p38, JNK, p-JNK, ERK1/2, p-ERK1/2, p65, p-p65, Bcl-2, cleaved-caspase 3, S100A9, Bax, p53, IL-6, TLR4, NOD2, and GAPDH. The membranes were washed three times and incubated again with the horseradish peroxidase (HRP) conjugated goat secondary antibodies (Beyotime, Haimen, Jiangsu, China). Then, the membranes were immersed with HRP substrate (Millipore Corporation, Billerica, USA) and signals were detected with the ChemiDoc XRS Imager (Bio-Rad, USA).

### Quantitative real-time RT-PCR

According to the manufacturer’s instructions, total RNA was extracted with TRIzol reagent (Ambion, USA) and quantified by a NanoDrop ND-2000 spectrophotometer (Thermo Fisher). After quality check, mRNA was reverse-transcribed to cDNA using the RevertAid First Strand cDNA Synthesis Kit (Thermo Scientific). SsoAdvanced™ Universal SYBR® Green (Bio-Rad) was used for qPCR measurement. Standard curves were constructed with the CFX96™ real-time PCR detection system (Bio-Rad). The cycling conditions for the qPCR were as follows: 95 °C for 3 min, followed by 37 cycles of 95 °C for 30 s, 55 °C for 30 s, and 72 °C for 30 s. The relative expression levels of genes were calculated using the 2^−ΔΔCt^ method based on the threshold cycle (C_t_) value.

### Cytokine assays

Colon tissue was homogenized in tissue protein extraction reagent. The homogenized tissue was centrifuged at 12,000 *g* for 10 min. The supernatant was collected and stored at − 80 °C until further analysis. TNF-α, interleukin-6 (IL-6), and interleukin-1β (IL-1β) in the supernatant were analyzed using ELISA kits according to the manufacturer’s instructions.

### Ethynyl-2′-Deoxyuridine (EdU) cell proliferation assay

The proliferation of colon cells was detected by the BeyoClick™ EdU-488 Kit (Beyotime, Haimen, Jiangsu, China). Briefly, 4 h before rats were sacrificed, they were injected intraperitoneally with 5 mg/kg EdU solution and then the colon was collected and rinsed with saline. Colon samples were then processed using the BeyoClick™ EdU-488 kit according to the manufacturer’s instructions. The EdU positive cells were detected with a confocal laser scanning microscope (LSM 710, ZEISS, Germany).

### Apoptosis detection assay

To further define the apoptotic cells in rat’s colon, the *In Situ* TUNEL Apoptosis Detection Kit (Nanjing Jiancheng Bioengineering Institute, Nanjing, China) was used to detect the apoptotic cells according to the manufacturer’s instructions. Briefly, rats were sacrificed after the colitis experiment. Then the colon samples were collected and stained with the detection kit. Terminal deoxynucleotidyl transferase (TdT) was used to label DNA strand breaks. Fluorescein labels incorporated in nucleotide polymers were detected by fluorescence microscopy and the percentage of TUNEL-positive cells was counted.

### Immunofluorescence assay

The protein levels of CD11b and S100A9 in colon tissues were detected by immunofluorescence staining. The colon sections were infiltrated with 0.5% Triton X-100 in PBS, blocked with 3% BSA, incubated overnight at 4 °C with FITC-anti-CD11b (1:100, Thermo Fisher Scientific, USA) or FITC-anti-S100A9 (1:100, Cell Signaling Technology) antibodies. The sections were counterstained with a secondary antibody for 1 h. The immunofluorescent cells were visualized and digital images were captured using a confocal laser scanning microscope (LSM 710, ZEISS, Germany).

### MPO activity

For measurements of MPO activity, colon samples were homogenized in 1:10 (w/v) of 50 mM PBS (pH 6.0) using a Polytron homogenizer. The homogenate was centrifuged at 12,000 rpm for 10 min. Then, MPO activity of homogenate was detected using a commercially available ELISA kit (Nanjing Jiancheng Bioengineering Institute, Nanjing, China) according to the manufacturer’s instructions. MPO activity was normalized by the weight of total sample and expressed as U/g tissue.

### Cell culture and treatment

Caco-2 cell line was purchased from Shanghai Cell Bank of Chinese Academy of Sciences (Shanghai, China) and routinely maintained in DMEM (Gibco, USA) supplemented with 10% fetal bovine serum (Gibco, USA), 100 µg/ml streptomycin and 100 U/ml penicillin (Gibco, USA).

Cells were cultured at 37 °C with 5% CO_2_. For all experiments, Caco-2 cells were treated with vehicle or 1 µg/ml LPS or recombinant human S100A9 protein for 1 h, and then treated with different concentration of anemoside B4. At 6 h or 24 h after treatment of anemoside B4, total protein samples were extracted from cells and prepared for qPCR or Western blotting experiments.

### Data and statistical analysis

Statistical analysis was performed using SPSS 16.0 statistical analysis software. Data were expressed as the mean ± SD. All statistical analyses were performed with a threshold for significance (alpha) set at 0.05. *P* < 0.05 was considered as statistically significant except the quantitative proteomics analysis. Two-way ANOVA followed by Dunnett’s multiple comparisons test was used to analyze the body weight change and DAI scores in the animal experiments. One-way ANOVA followed by Dunnett’s multiple comparison test was used for other data except the body weight change and DAI scores.

## Results

### Anemoside B4 ameliorated TNBS-induced colitis in rats

The TNBS hapten, given as one or two enemas with ethanol as a carrier to disrupt epithelial integrity, induces acute inflammation and colitis in the rats with Crohn’s colitis-like transmural tissue damage [21]. In this study, TNBS-induced severe acute colitis model was used to determine the pharmacological activity of anemoside B4. The doses of anemoside B4 were chosen according to our preliminary tests on LPS-induced inflammation and acetic acid-induced analgesia experiments [11]. Mesalazine was orally administrated to rats as the positive control [21].

**Fig. 1:**
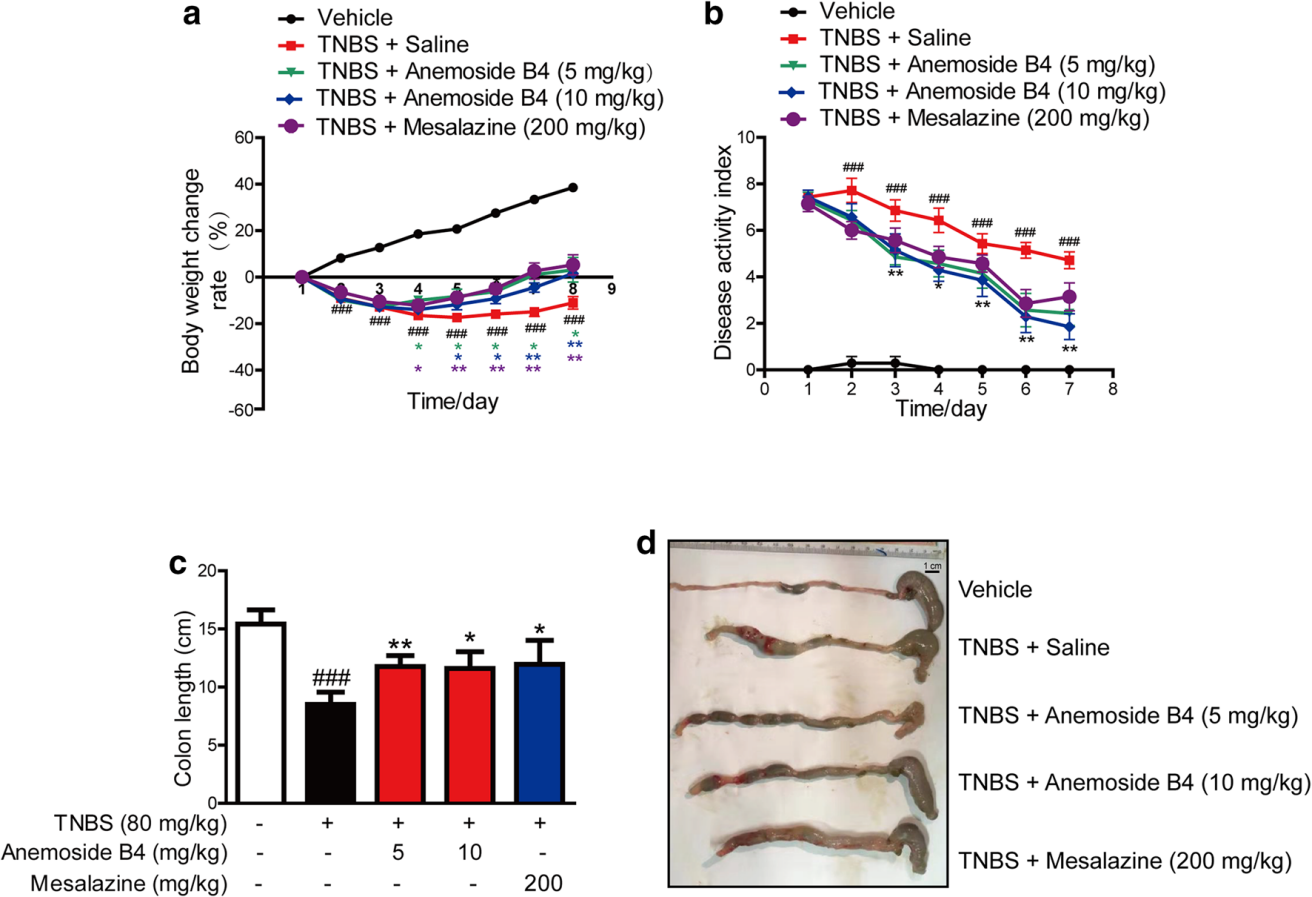
Anemoside B4 ameliorates TNBS-induced colitis in rats. Rats were orally administrated with 80 mg/kg TNBS for one day and then were continuously injected with anemosdie B4 at the doses of 5 and 10 mg/kg twice per day for 7 days. Mesalazine was administrated consecutively i.g. at the dose of 200 mg/kg once per day for 7 days. Rats were sacrificed on the 8th day after colitis induction. **a** Body weight of each group was recorded during the progression. **b** DAI of each group during the disease process. **c** Colon length of rats from different groups. **d** Representative images of colons at the end of the experiment. Scale bar, 100 µm. Results in (**a**–**c**) are presented as mean ± SD (n = 7). Body weight change and disease activity index, two-way ANOVA followed by Dunnett’s multiple comparisons test. ^###^: *P* < 0.001, vs. vehicle group, *: *P* < 0.05, **: *P* < 0.01, ***: *P* < 0.001 vs. TNBS + saline group

In the model group, we observed IBD-like colitis, diarrhoea and bloody mucopurulent stools. Compared with the vehicle group, TNBS-treated rats showed a significantly body weight loss. However, both anemoside B4 and mesalazine reduced body weight loss during disease progression (Fig. 1a). Besides, TNBS induced a significant increase in DAI score, an important marker of IBD. In contrast, anemoside B4 ameliorated TNBS-induced DAI score in a dose-dependent manner and mesalazine was slightly less effective (Fig. 1b). TNBS treatment induced a significant decrease in colon length. Furthermore, treatment with anemoside B4 ameliorated TNBS-induced colon length shortening and mesalazine was slightly less effective (Fig. 1c and d). These results demonstrated that inhibitory effect of anemoside B4 on colitis is slightly more effective than mesalazine.

### Anemoside B4 suppressed TNBS-induced inflammation

**Fig. 2:**
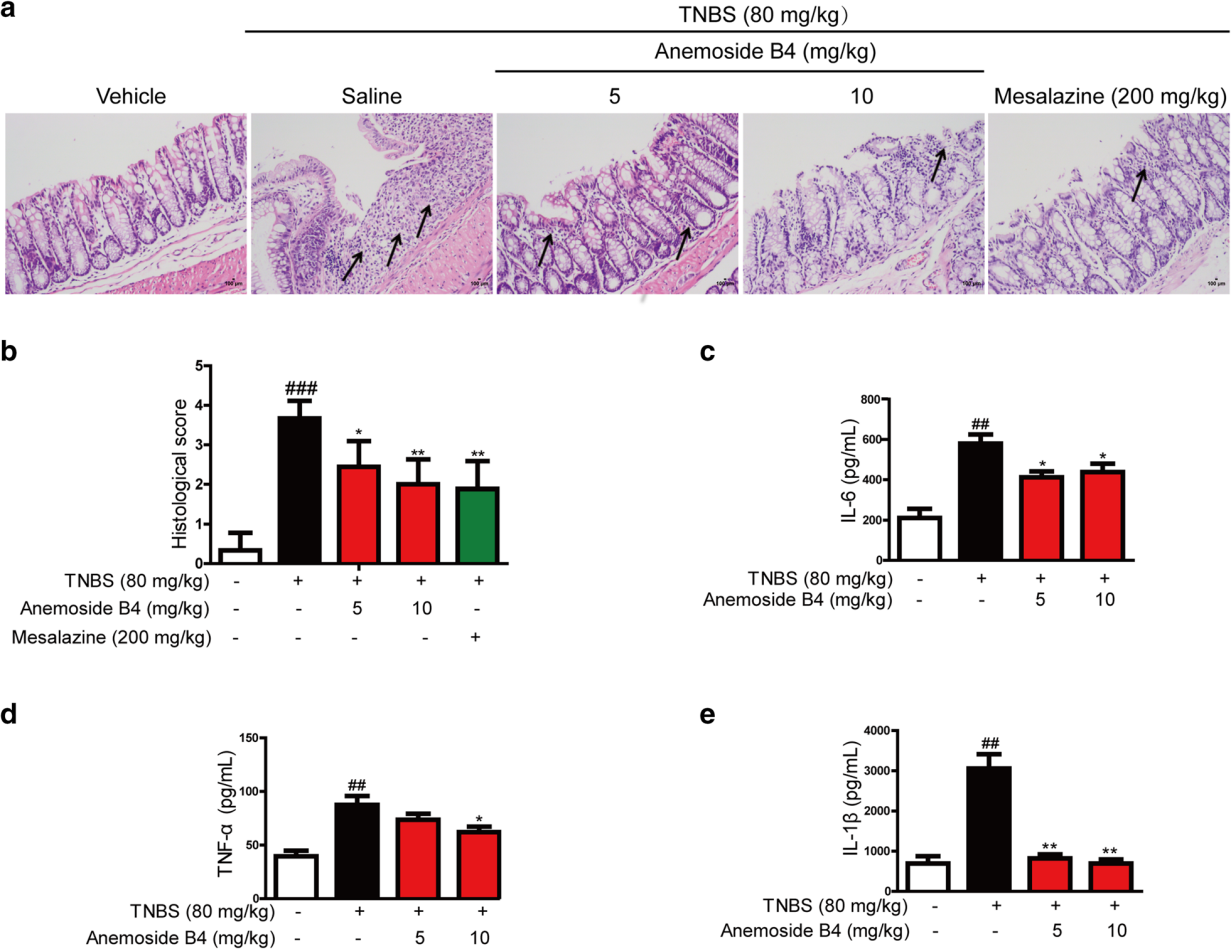
Anemoside B4 ameliorates inflammatory response of TNBS-induced colitis in rats. **a** Serial sections of colon tissues were stained with hematoxylin and eosin (H&E). Black arrows indicated the apoptosis cells. Colon histology was examined by H&E staining of paraffin-embedded sections in vehicle and TNBS-treated rats on the 8th day after TNBS administration. Scale bars: 100 µm. **b** Histological scores. **c** IL-6, **d** TNF-α, and **e** IL-1β were measured by ELISA. Data were shown as mean ± SD (n = 3). ^##^: *P* < 0.01, vs. vehicle group. *: *P* < 0.05, **: *P* < 0.01 vs. TNBS group

H&E staining showed that the colon tissues of the rats in TNBS-induced groups had pathological structures, including obvious infiltration of inflammatory cells, epithelial cell damage and apoptosis. There was marked difference in pathological score between the normal group and TNBS group. However, anemoside B4 relieved all pathological changes and score in a dose-dependent manner (Fig. 2a and b).

TNBS-induced acute colitis maintains many pathological features of human colitis, especially increasing type I inflammatory response and helper T cell 1 (Th1)-related cytokines, such as TNF-α or interferon-γ (IFN-γ). During inflammation, monocytes are recruited to inflamed sites and lymphoid tissues, where macrophage differentiation plays an important role during the beginning and resolution of inflammatory processes [22]. Therefore, we measured the effect of anemoside B4 on immune cell infiltration and inflammatory cytokine expression in rats with TNBS-induced acute colitis. Compared with the rats in vehicle group, those exposed to TNBS had significantly (*P* < 0.05) increased levels of colonic pro-inflammatory cytokines and chemokines, including IL-6, TNF-α, and IL-1β. However, anemoside B4 significantly reduced the levels of these colonic pro-inflammatory cytokines (Fig. 2c–e).

### Anemoside B4 suppressed TNBS-induced proliferation and apoptosis

**Fig. 3:**
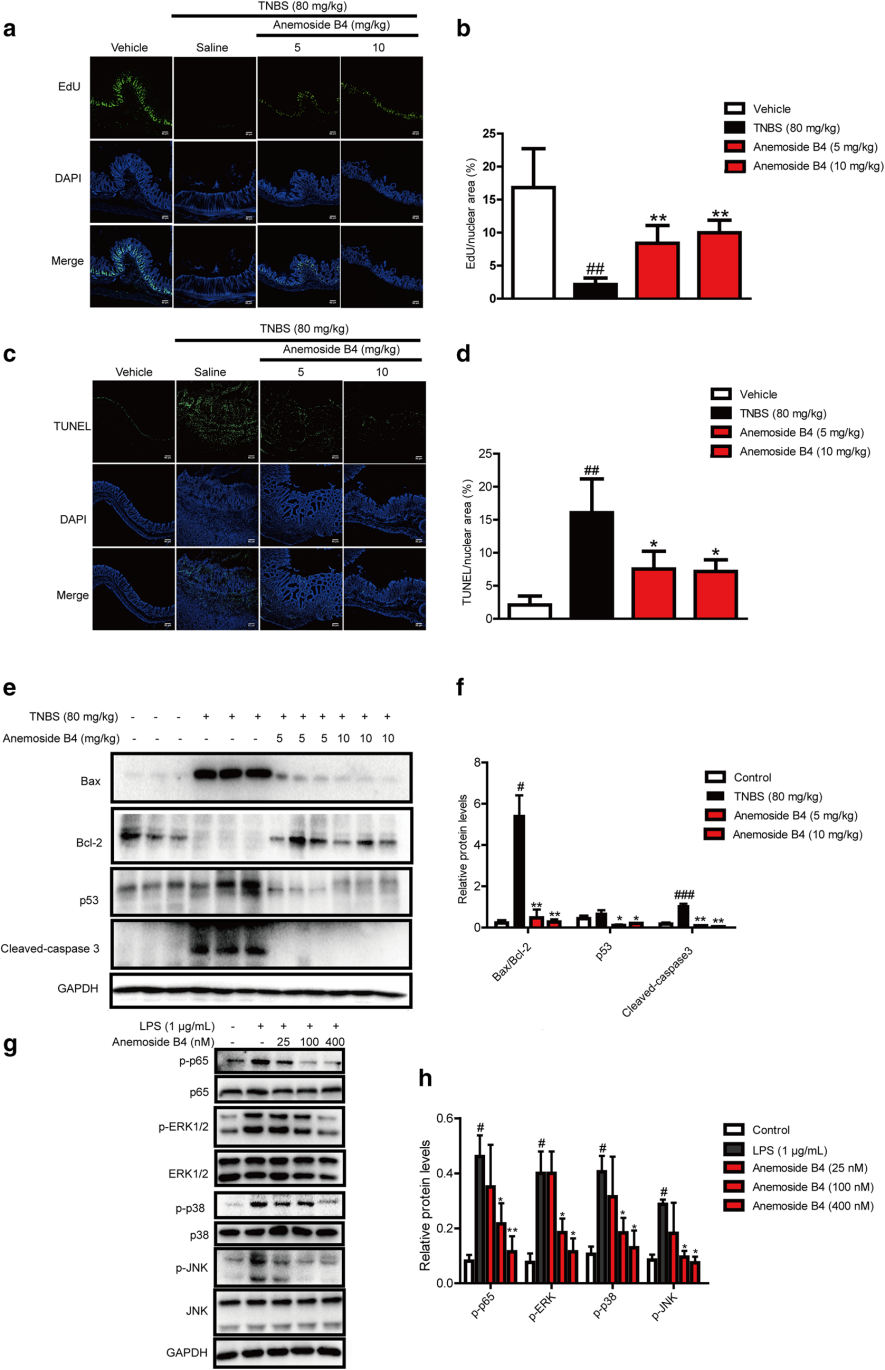
Inhibitory effect of anemoside B4 on the proliferation, apoptosis or inflammation induced by TNBS or LPS. **a **Sections of colon tissue (n = 3) were immunostained with Edu and observed by confocal laser-scanning microscope. **b** Fluorescence intensity of Edu stained points. **c** Sections of colon tissue were immunostained with TUNEL and observed by confocal laser-scanning microscope. **d** Fluorescence intensity of TUNEL stained points. **e** p53, Bcl-2, Bax and cleaved caspase 3 was measured by Western blotting. **f** Statistical analysis of p53, Bcl-2, Bax and cleaved caspase 3 protein level. **g** LPS-induced NF-κB activation. Caco-2 cells were treated with anemoside B4 as indicated concentration and LPS (1 µg/ml) for 60 min. Cells were harvested and total cell extracts were prepared. p-ERK, p-JNK, p-p38 and p65 were detected by Western blotting analysis. Total ERK, JNK, p38, p65 and GAPDH were used as internal standards. Each experiment was repeated for three times. **h** Western blotting analysis of proteins in the MAPK/NF-κB pathway. ^#^: *P* < 0.05, ^##^: *P* < 0.01, ^###^: *P* < 0.001 vs. control group. *: *P* < 0.05, **: *P* < 0.01 vs. TNBS group

Next, we explored the effects of anemoside B4 on the proliferation and apoptosis induced by TNBS. Normal colonic epithelial cells have regenerative proliferation indicated by green EdU stain. More EdU stained spots were observed in vehicle samples. However, TNBS decreased EdU stained spots, suppressing the ability of regenerative proliferation. However, anemoside B4 partially restored the ability of regenerative proliferation (Fig. 3a–b).

It has been previously reported that TNBS-induced colitis is associated with apoptosis of the colonic epithelial cells and its toxicology is thought to be related to the induction of apoptosis and the destruction of the intestinal mucosal barrier [23, 24]. In IBD, there are high levels of apoptosis in the intestinal epithelium of patients [25, 26]. The increased number of apoptotic epithelial cells and elevated Bax but attenuated Bcl-2 during active colitis may lead to a defective epithelial barrier and result in pathogenic microorganism infiltration.

In the present study, TUNEL assay was carried out to detect apoptotic cells. More TUNEL stained spots were observed in the colon tissue obtained from TNBS-induced colitis rats. Anemoside B4 treatment significantly decreased the TUNEL stained spots (Fig. 3c, d). Furthermore, the relative expression levels of apoptosis associated proteins were detected by Western blotting analysis. Consistent with previous reports, we found that the TNBS group had markedly up-regulated Bax, p53 and cleaved caspase 3 but down-regulated Bcl-2 in the colon. In contrast, anemoside B4 significantly increased Bcl-2/Bax ratio and reduced cleaved caspase3 and p53 protein levels in a dose dependent manner, suggesting that anemoside B4 could reduce colitis apoptosis in colons (Fig. 3e, f). These observations obviously indicated that anemoside B4 was effective in the treatment of TNBS-induced acute colitis in rats.

### Anemoside B4 inhibited NF-κB activation induced by LPS in colonic epithelial cells

The above experiment data showed that anemoside B4 had anti-inflammatory activity and inhibited inflammatory cytokine secretion induced by colitis. It is well-known that NF-κB plays a critical role in inflammation in various human diseases including IBD and in animal disease models. Activated NF-κB pathway promotes the expression of various pro-inflammatory genes and influences the course of mucosal inflammation [27].

To explore the mechanism of the anti-inflammatory activity of anemoside B4, we investigated the effect of anemoside B4 on the activation of the NF-κB signaling pathway as it had been documented to be a key signaling pathway in inflammation disease. As expected, the level of p-p65/p65 is increased upon LPS treatment, while it was significantly reduced by anemoside B4 (Fig. 3g, h). In addition, LPS activated NF-κB upstream signaling pathway, including p-p38, p-JNK, and p-ERK in colonic epithelial cells, while their levels were similarly reduced by anemoside B4 (Fig. 3g, h).

### Proteomic identification of the differentially regulated proteins by anemoside B4

The above studies showed that 10 mg/kg anemoside B4 remarkably diminished the TNBS-induced colitis and the therapeutic effect was comparable to that of mesalazine. Therefore, in the following experiments, anemoside B4 was injected at the dose of 10 mg/kg.

To further investigate the mechanisms of the therapeutic effect of anemoside B4 on TNBS-induced colitis, we performed a label-free quantitative proteomic analysis to identify the differentially expressed proteins in the colon samples from TNBS-induced group and anemoside B4-treated group.

**Fig. 4:**
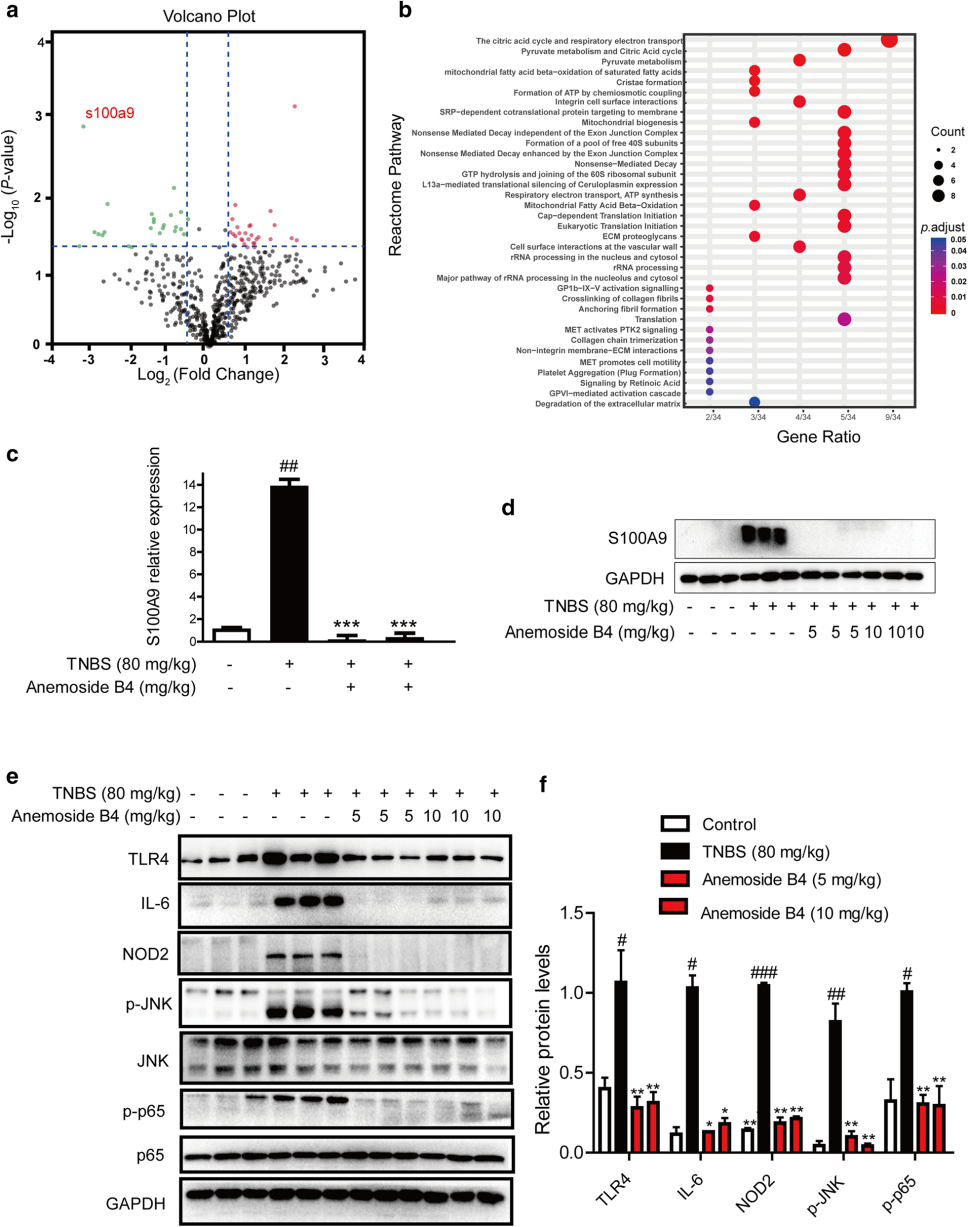
Quantitative proteomic and biochemical analyses identify the differentially regulated proteins by anemoside B4 upon TNBS-induced colitis colon tissue from rats. Rats were orally administrated with 80 mg/kg TNBS. Anemoside B4 was continuously injected i.p at the doses of 10 mg/kg twice a day for 8 days. Rats were sacrificed on day 8 after colitis induction. **a** The volcano plot for the MS identified 604 proteins in colon tissue. Each point showed the log_2_ (Fold change) in the *x* axis vs. their corresponding -log_10_ (*P*-value) in the *y* axis. The filter boundaries were set so that a fold change above 1.5, or a fold change below 0.67, were considered significant, and a *P*-value of 0.05 was used as the cutoff value. The horizontal dotted line indicates the cutoff *P*-value of 0.05 and all points above this line have *P* < 0.05. The black points indicate 591 quantified unaltered proteins. The green points and red points indicated the down-regulated and up-regulated proteins, respectively. The circle indicated the proteins used for the biochemical validation. **b** The biological processes of the differentially regulated proteins analyzed by the online STRING database. **c** Quantification of S100A9 mRNA expression. **d** Western blotting analysis of S100A9 protein level. **c**, **d** Data were shown as mean ± SD (n = 3). ^##^: *P* < 0.001 vs. vehicle group; ***: *P* < 0.001 vs. TNBS group. (E) Proteins in the downstream or upstream of S100A9 signaling pathway were detected by Western blotting in colon tissues (n = 3). **f** Analysis of S100A9 protein level. ^#^: *P* < 0.05, ^##^: *P* < 0.01, ^###^: *P* < 0.001 vs. control group. **: *P* < 0.01 vs. TNBS group

In total, we identified 604 proteins and quantified 591 proteins, whose ratios were shown in the volcano plot. Among the quantified proteins, 56 protein groups with fold change > 1.50 or < 0.67 and *P* < 0.05 were marked with red (upregulation) or green (downregulation) dots in the anemoside B4-treated group compared with the TNBS-induced group (Fig. 4a).

Next, the biological processes that the 56 proteins participated in were analyzed through the online STRING database. The result showed that the differentially regulated proteins were mainly clustered in the tricarboxylic acid (TCA) cycle and respiratory electron transport chain (Fig. 4b). S100A9 protein is one of the obviously reduced proteins and mostly affected by anemoside B4 (Fig. 4a).

Western blotting and qPCR analysis revealed that both the protein and mRNA levels of S100A9 were significantly increased in colon tissues treated by TNBS and these increases could be attenuated by the further treatment with anemoside B4 (Fig. 4c-d), consistent with the proteomic data.

### Anemoside B4 suppressed S100A9/MAPK/NF-κB downstream signaling pathway in vivo

S100A9 interacts with S100A8 to form heterodimers of S100A8/S100A9, which bind to TLR4. The downstream activation of JNK and NF-κB occurs through MyD88-induced activation in a TLR4 dependent manner, thereby promoting inflammation or apoptosis. Therefore, we examined the activation of S100A9/MAPK/NF-κB signaling pathway in colonic homogenate to explore the further molecular evidence of the anti-inflammatory effects of anemoside B4. As shown in Fig. 4e, TNBS induction significantly increased the expression of TLR4 protein in rat colon, which was significantly reduced by the treatment of anemoside B4. We further measured the expressions of some key proteins involved in the downstream pathway of S100A9, such as p-NF-κB/p-p65, p-JNK, effector molecules IL-6 and NOD2. All of them were significantly increased in rat colons after TNBS challenge. However, anemoside B4 could reduce the increased expressions (Fig. 4e, f). These results demonstrated that anemoside B4 could inhibit the TNBS-induced activation of S100A9/MAPK/NF-κB pathway in rat colons.

### Anemoside B4 inhibited the recombinant S100A9 protein-stimulated downstream signaling pathways in colonic epithelial cells

**Fig. 5:**
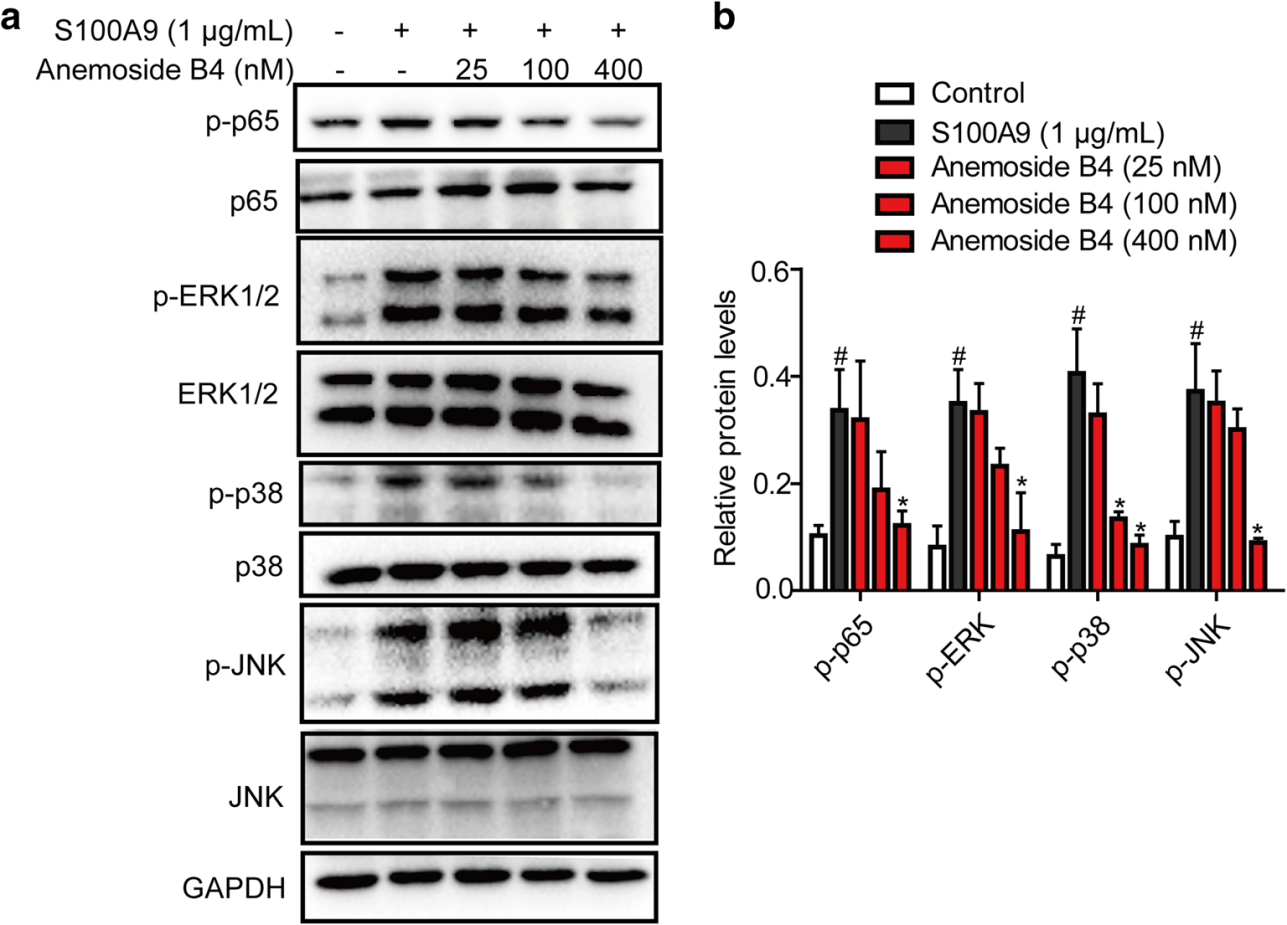
Anemoside B4 attenuates the effect of S100A9 recombinant protein on the phosphorylation and activation of NF-*κ*B and MAPKs in Caco-2 cells. Human Caco-2 cells were treated with S100A9 recombinant protein (1 µg/mL) for 1 h, and cell were treated with anemoside B4 for 6 h. **a** Phosphorylation of p65, ERK1/2, JNK1/2 and p38 MAPK and total proteins were analyzed by Western blotting. **b** Analysis of S100A9 and its downstream protein level. Data were shown as mean ± SD (n = 3). Each experiment was repeated for three times. ^#^: *P* < 0.05 vs. control group. *: *P* < 0.05 vs. S100A9 group

Recombinant S100A9 protein stimulated the expressions of its downstream target genes including TNF-α and IL-1β in macrophages, while anti-S100A9 antibody significantly blocked this effect [28]. To evaluate whether S100A9 is a key factor in the effect of anemoside B4, S100A9 recombinant protein was used to treat colonic epithelial Caco-2 cells. The cell lysate was collected 24 h after treatment of recombinant S100A9 protein. As shown in Fig. 5a, S100A9 activated the phosphorylation of NF-κB/p65. In contrast, anemoside B4 inhibited this activation. An important upstream component of NF-κB signaling is the MAPK signaling pathway, which comprises of p38, JNK and ERK1/2. MAPK activation also induces phosphorylation of NF-κB/p65 [29]. In our study, S100A9 increased the phosphorylation of p38, ERK1/2 and JNK. However, treatment with anemoside B4 ameliorated this increase (Fig. 5a, b). Hence, we could convincingly conclude that anemoside B4 mediated its protective role by down-regulating S100A9 signaling pathway.

### *Anemoside B4 inhibited the recruitment of inflammatory cells to colon*

The recruitment of inflammatory cells to the site of infection or inflammation represents an important process in IBD. Neutrophils are often the first immune cells recruited to the site of inflammation and play a key role in combatting microbial invasion [30].

**Fig. 6:**
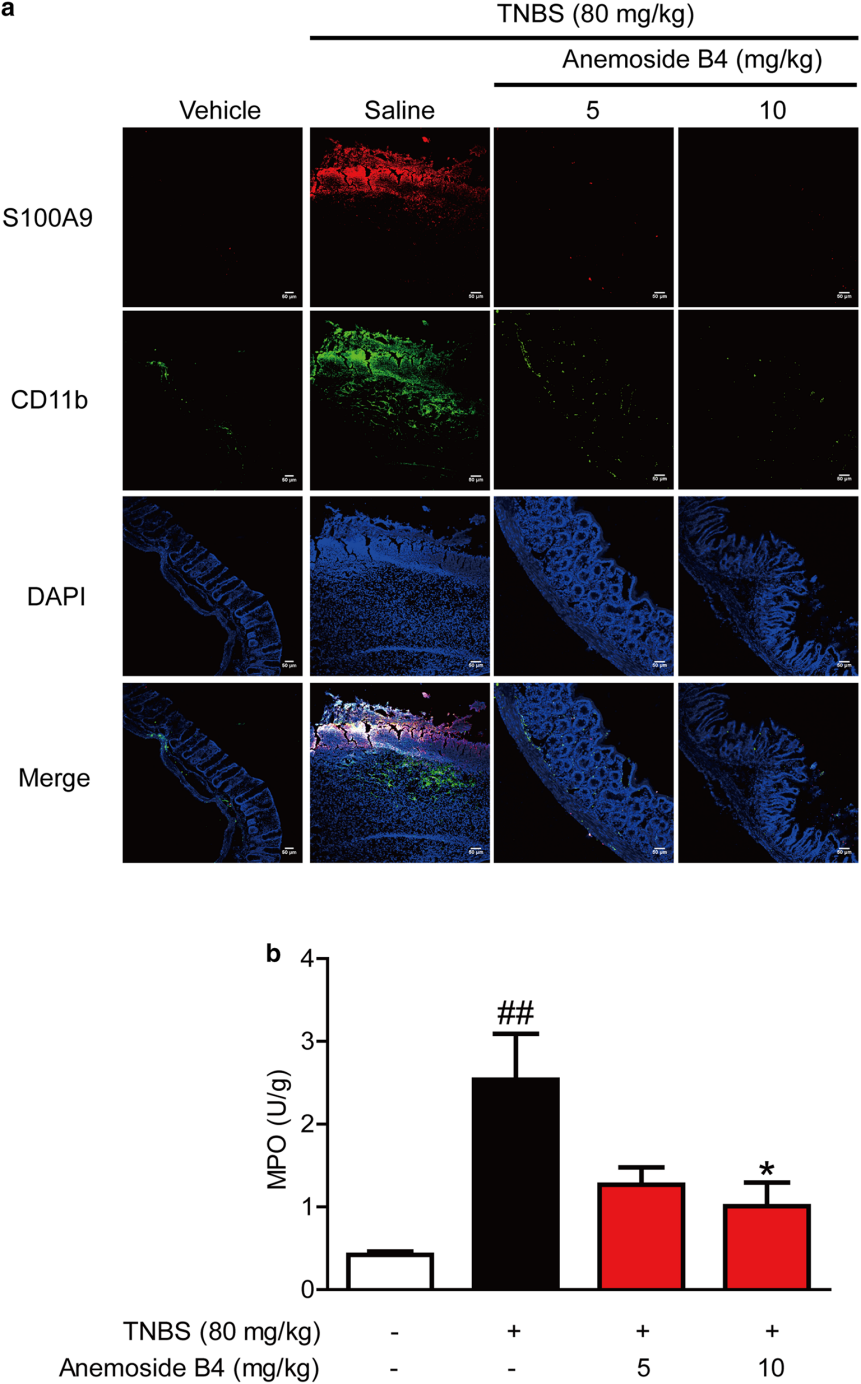
Anemoside B4 attenuates the recruitment of inflammatory cells to colon. **a** Sections of colon tissue were immunostained with FITC-S100A9 (red) and FITC-CD11b (green) and the immunofluorescence was detected by confocal laser-scanning microscope. Shown were data from three rats in one experiment (n = 3 per group). (B) MPO in colon tissues (U/g) was determined by ELISA kits. Data were shown as mean ± SD (n = 3)

To evaluate the effect of anemoside B4 on inflammatory infiltration in a rat model of intestinal inflammation, a fluorescence labelled antibody against rat CD11b was used to measure inflammatory recruitment and infiltration into the rat intestine. Compared to non-colitic control rats, colitis animals showed a significantly elevated number of CD11b positive cells in the intestinal wall (Fig. 6a). By contrast, anemoside B4 significantly decreased the number of positive cells in colon tissue of the TNBS-induced colitis.

In inflammation tissue, a few types of cells can secret S100A9, such as intestinal epithelial cells, neutrophils, monocyte, and macrophage. However, it is not clear where S100A9 comes from after the treatment with anemoside B4.

To answer this question, we measured the localization of S100A9. The data showed that there were more S100A9 positive cells in colon tissue from colitic rats than from vehicle rats. In contrast, anemoside B4 significantly decreased S100A9 positive cells in colon tissue. It is interesting that S100A9 localization is consistent with the location of CD11b positive cells. These data indicated that CD11b positive cells are the major source of S100A9.

Additionally, the concentration of MPO, which reflects both neutrophil numbers and activity, was greatly elevated in colonic tissue. By contrast, anemoside B4 significantly decreased the concentration of MPO in colon tissue (Fig. 6b). These data indicated that anemoside B4 significantly inhibited inflammation cell recruitment, especially in neutrophils.

## Discussion

In this paper, we reported the in vitro and in vivo therapeutic effect of anemoside B4 on colitis and explored its anti-inflammatory and anti-apoptotic activities. Our experiments indicated that anemoside B4 ameliorated the apoptosis, inflammatory cell infiltration, and pro-inflammatory cytokine production in the TNBS-induced colitis. Furthermore, we found that the molecular mechanism of action of anemoside B4 involved in the S100A9/MAPK/NF-κB signaling pathway through quantitative proteomics and biochemical experiments. Our previous study showed that anemoside B4 has low toxicity without influencing body weight and function of hepatitis and kidney in mice [11]. Taken together, these studies indicated that anemoside B4 might have therapeutic potential for IBD.

IBD is a chronic inflammatory disease of the gastrointestinal tract characterized by breakdown of the epithelial barrier and disruption of intestinal homeostasis. Emerging experimental and clinical data have indicated that pro-inflammatory cytokines such as TNF-α, IFN-α and IL-6 play crucial roles in pathogenesis of colitis [31, 32]. In this study, anemoside B4 exerted its anti-inflammatory effect by inhibiting the secretion of pro-inflammatory factors such as IL-1β, IL-6 and TNF-α in colon. These are consistent with the results of previous studies [11, 33].

Numerous studies have identified that apoptosis in intestinal epithelial cells is driven by increased cytokine activity such as TNF, IL, and IFN family proteins [34]. Increased apoptosis of intestinal epithelial cells can also disrupt intestinal mucosal integrity and barrier function, eventually leading to inflammation [23]. Elucidating the molecular mechanism how to efficiently decrease colonic epithelial cell apoptosis and how to repair mucosal tissues has become the focus of therapy of UC [35]. Our results indicated that colitis rats had more apoptotic cells, evidenced by the increased Bax and cleaved caspase3 but reduced anti-apoptosis protein Bcl-2. Treatment with anemoside B4 decreased the number of apoptotic cells and moderated the expression levels of these apoptosisrelated proteins. Thus, anemoside B4 might protect the integrity of the intestinal barrier by inhibiting apoptosis of intestinal epithelial cells.

In order to elucidate the mechanism by which anemoside B4 attenuates colitis, we performed a label-free quantitative proteomic study and identified 56 proteins regulated by anemoside B4. Among them, S100A9 might play a vital role in the regulation of the biological effect of anemoside B4.

S100A9 is an intracellular calcium-binding protein belonging to the S100 family, which was originally discovered as an immunogenic protein expressed and secreted by neutrophils [36]. Besides neutrophils and monocytes, S100A8 and S100A9 can also be induced in keratinocytes and epithelial cells under inflammatory conditions [37, 38]. Growing evidence shows that S100A9 has a dual role in the inflammation response, which is strongly upregulated in trauma, infections, heat, stress, and many other inflammations. S100A9 stimulates all members of the MAPK cascades, including p38, ERK, and JNK and activates NF-κB through TLR4 or RAGE receptor [39]. In intestinal inflammation, S100A9 is an effector molecule that enhances TLR signaling and recruits granulocytes. Thus, the blockade of this molecule could ameliorate disease severity in colitis [28]. In present study, anemoside B4 reduced the activation of S100A9/MAPK/NF-*κ*B signaling pathway in vitro and in vivo. Anemoside B4 significantly decreased TLR4 expression and its downstream signaling molecules, including the phosphorylation of NF-κB/p65, JNK, and expression of effector molecule IL-6. Further, experiments with colonic epithelial Caco-2 cells treated with recombinant S100A9 protein found S100A9 activated NF-κB/p65. An important upstream component of NF-κB is the MAPK signaling pathway. MAPK activation also induces NF-κB/p65 activation. Anemoside B4 inhibited MAPK activation which was stimulated by the recombinant S100A9 protein.

S100A8 and S100A9 comprise ~ 40% of the cytosolic proteins of neutrophils and their intracellular expression has been associated with calcium sensing [40]. Infiltration of neutrophils into colonic mucosa has been associated with the severity of IBD. Neutrophil in the intestinal mucosa constitutes a prominent inflammatory component of active UC. S100A9 is not only synthesized and secreted by neutrophils, but also binds to neutrophils and activates intracellular signaling that promote neutrophil migration [41]. Neutrophils are often the first type of immune cells recruited to the site of inflammation and play a key role in combatting microbial invasion. When these pathways become dysregulated, extensive tissue damage and development of chronic disease might occur. The role of neutrophils in the pathogenesis of IBD remains controversial and possibly differs between UC and CD. Currently, there is no therapeutic strategy to selectively target neutrophils [42]. Monitoring neutrophil trafficking to the site of inflammation would be helpful for understanding their contribution to the pathogenesis of disease. This would help to assess the anti-inflammatory effects of therapeutic drugs. Further, our immunofluorescence experiment found that S100A9 is located on the surface of colon. TNBS induces myeloid cells, especially neutrophils, to migrate into damaged colon mucosa. S100A9 shares the same location of neutrophils in tissue. These data suggested that anemoside B4 inhibited the migration of neutrophils to colon in colitis.

Then we measured the activities of MPO in the colon tissues of rats in each group. MPO is present in primary granules for medially during neutrophil maturation and liberated in copious amounts by activated neutrophils to amplify the inflammatory response by inducing oxidative modification of proteins and lipids. Its activity linearly correlates to neutrophil infiltration, a marker of infiltration of neutrophil, in inflamed tissue [43]. Confirmatory, colonic MPO levels, which are correlated with the number of neutrophils, were more significantly increased in colon tissue obtained from colitic rats than from non-colitic rats [44, 45]. When compared with that of the TNBS-induced group, treatment with anemoside B4 significantly suppressed the elevated MPO activity. Furthermore, our immunofluorescence experiment found that S100A9 is distributed on the surface of colon. Myeloid cells migrated into inflammatory colon mucosa induced by TNBS. S100A9 is located in the same place in tissue. These data demonstrated that anemoside B4 inhibited the migration of myeloid cells and the expression of S100A9. Based on these experiments, our data suggested that anemoside B4 inhibited neutrophil accumulation. Further experiments are required to elucidate the underlying molecular mechanism.

## Conclusions

**Fig. 7:**
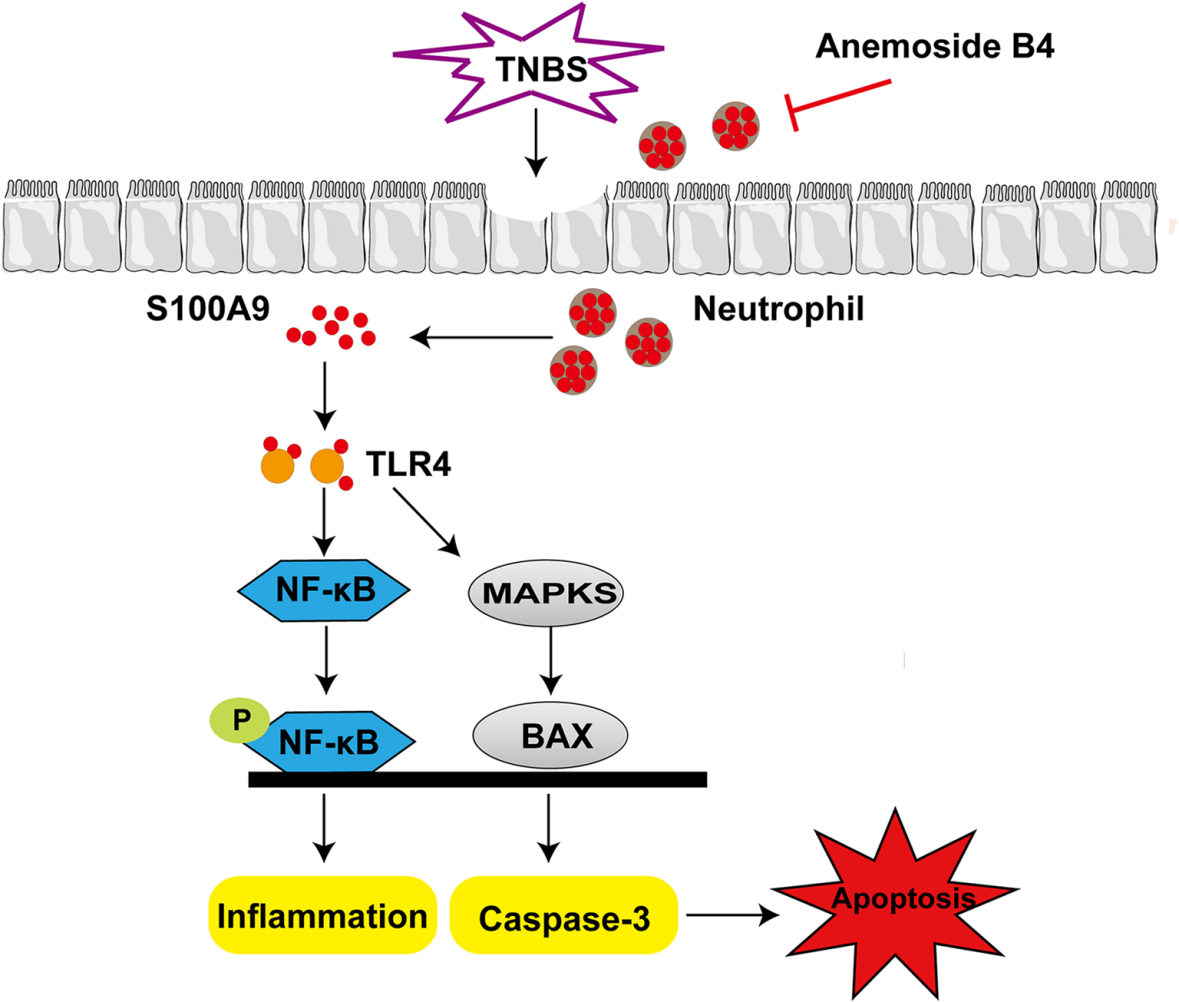
The schematic model for the mechanism of action of anemoside B4 in the treatment of colitis

In summary, our study demonstrated that anemoside B4 exhibited significant protective effects on TNBS-induced colitis via suppressing the S100A9/MAPK/NF-κB signaling pathway and inhibited neutrophil recruitment and activation (Fig. 7).

## Data Availability

Not applicable.

## Abbreviations

CD: Crohn’s disease
DAI: Disease activity index
Edu: Ethynyl-2’-deoxyuridine
IBD: Inflammatory bowel disease
IFN-γ: Interferon-γ
IL-1β: Interleukin-1β
IL-6: Interleukin-6
MAPK: Mitogen-activated protein kinases
MPO: Myeloperoxidase
MyD88: Myeloid differentiation factor 88
NF-κB: Nuclear factor kappa-light-chain-enhancer of activated B cells
RAGE: Receptor for advanced glycation end products
TCA: Tricarboxylic acid
TLR4: Toll-like receptor 4
TNBS: 2, 4, 6-Trinitrobenzene sulfonic acid
TNF-α: Tumor necrosis factor
TUNEL: Terminal deoxynucleotidyl transferase dUTP nick-end labeling
UC: Ulcerative colitis
